# The Brain Gene Registry: a data snapshot

**DOI:** 10.1186/s11689-024-09530-3

**Published:** 2024-04-17

**Authors:** Dustin Baldridge, Levi Kaster, Catherine Sancimino, Siddharth Srivastava, Sophie Molholm, Aditi Gupta, Inez Oh, Virginia Lanzotti, Daleep Grewal, Erin Rooney Riggs, Juliann M. Savatt, Rachel Hauck, Abigail Sveden, the Brain Gene Registry Consortium, Melissa Wasserstein, Mustafa Sahin, Michael F. Wangler, Robert Schultz, Andrea Gropman, Constance Smith-Hicks, Len Abbeduto, Kendell German, Leann Smith DaWalt, Jeffrey L. Neul, Steven U. Walkley, Eric A. Storch, Rodney Samaco, Kosuke Izumi, Juhi Pandey, Seth I. Berger, Julie S. Cohen, Suma Shankar, Dan Doherty, Sonal Mahida, Kira A. Dies, Megan Clarke, Alexa Taylor, Madison Berl, Ryan German, Christina Nguyen, Holly K. Harris, Amanda Hut, Vanessa Gomez, Carrie L. Arneson, Isaac Horn, Gabriel Damon Lavezzi, Diane Grypp, Devinae McNeil, Cailin White, Julie Rusyniak, Abigail Moradel Higareda, Paul Deppen, Anna Bican, Madeline Rockouski, Emily Schneider, Madeline Thompson, Jessica Kinard, Brittany Minor, John N. Constantino, Joseph Piven, Christina A. Gurnett, Maya Chopra, Heather Hazlett, Philip R. O. Payne

**Affiliations:** 1 Department of Pediatrics, Washington University School of Medicine in St. Louis, St. Louis, MO USA; 2 Institute for Informatics, Data Science and Biostatistics, Washington University School of Medicine in St. Louis, St. Louis, MO USA; 3 Department of Pediatrics, Albert Einstein College of Medicine, Bronx, NY USA; 4 Department of Neurology, Boston Children’s Hospital, Harvard Medical School, Boston, MA USA; 5 Rosamund Stone Zander Translational Neuroscience Center, Boston Children’s Hospital, Boston, MA USA; 6 Departments of Pediatrics and Neuroscience, Albert Einstein College of Medicine, Bronx, NY USA; 7 Department of Psychiatry, Washington University School of Medicine in St. Louis, St. Louis, MO USA; 8 Autism and Developmental Medicine Institute, Geisinger, Danville, PA USA; 9 Department of Genomic Health, Geisinger, Danville, PA USA; 10 Division of Behavioral and Mental Health, Departments of Psychiatry and Pediatrics, Children’s Healthcare of Atlanta, Emory University, Atlanta, GA USA; 11 The Carolina Institute for Developmental Disabilities, University of North Carolina, Chapel Hill, NC USA; 12 Department of Neurology, Washington University School of Medicine in St. Louis, St. Louis, MO USA

**Keywords:** Brain gene registry, Neurodevelopmental disorders, Electronic health records

## Abstract

Monogenic disorders account for a large proportion of population-attributable risk for neurodevelopmental disabilities. However, the data necessary to infer a causal relationship between a given genetic variant and a particular neurodevelopmental disorder is often lacking. Recognizing this scientific roadblock, 13 Intellectual and Developmental Disabilities Research Centers (IDDRCs) formed a consortium to create the Brain Gene Registry (BGR), a repository pairing clinical genetic data with phenotypic data from participants with variants in putative brain genes. Phenotypic profiles are assembled from the electronic health record (EHR) and a battery of remotely administered standardized assessments collectively referred to as the Rapid Neurobehavioral Assessment Protocol (RNAP), which include cognitive, neurologic, and neuropsychiatric assessments, as well as assessments for attention deficit hyperactivity disorder (ADHD) and autism spectrum disorder (ASD). Co-enrollment of BGR participants in the Clinical Genome Resource’s (ClinGen’s) GenomeConnect enables display of variant information in ClinVar. The BGR currently contains data on 479 participants who are 55% male, 6% Asian, 6% Black or African American, 76% white, and 12% Hispanic/Latine. Over 200 genes are represented in the BGR, with 12 or more participants harboring variants in each of these genes: *CACNA1A, DNMT3A, SLC6A1, SETD5*, and *MYT1L.* More than 30% of variants are de novo and 43% are classified as variants of uncertain significance (VUSs). Mean standard scores on cognitive or developmental screens are below average for the BGR cohort. EHR data reveal developmental delay as the earliest and most common diagnosis in this sample, followed by speech and language disorders, ASD, and ADHD. BGR data has already been used to accelerate gene-disease validity curation of 36 genes evaluated by ClinGen’s BGR Intellectual Disability (ID)-Autism (ASD) Gene Curation Expert Panel. In summary, the BGR is a resource for use by stakeholders interested in advancing translational research for brain genes and continues to recruit participants with clinically reported variants to establish a rich and well-characterized national resource to promote research on neurodevelopmental disorders.

**Supplementary Information:**

The online version contains supplementary material available at 10.1186/s11689-024-09530-3.

## Introduction

As next-generation sequencing for neurodevelopmental disorders (NDDs) grows, there is a major need to continually categorize variant-to-phenotype relationships, particularly for variants of uncertain significance (VUSs). NDDs are a group of conditions characterized by deficits in one or more developmental domain that manifest early during development, and result in functional impairments [1]. Examples of NDDs include global developmental delay, intellectual disability (ID), autism spectrum disorder (ASD), developmental and epileptic encephalopathy, and cerebral palsy (CP). Systematic meta-analyses and clinical practice guidelines support the use of broad unbiased sequencing tests (e.g., exome or genome sequencing) as a first-line approach with a diagnostic yield of 20–40% depending on the test, and type and severity of NDD [2–5]. One consequence of agnostic sequencing, whether by exome sequencing, genome sequencing, or sequencing panels that include a large number of genes, is the common occurrence of VUSs. As defined by the American College of Medical Genetics and Genomics (ACMG) [6], VUSs may apply to known human disease genes or candidate genes, defined as putative, but not yet established, human disease genes. The prevalence of VUSs identified by genome sequencing of individuals with NDDs is around 20–26% [7, 8]. VUSs are found in a higher proportion of individuals from historically underrepresented and minoritized backgrounds [9] and in those undergoing multi-gene panel testing [10].

To address this critical need to resolve VUSs and to better define the genotypic and phenotypic spectrum of genetic disorders, the Brain Gene Registry (BGR) was conceived to collect genotype and phenotype information on individuals with clinically identified variants in genes that have been linked to NDDs with varying levels of gene-disease validity evidence. These genes are referred to here as “brain genes.” The overarching goal of the BGR is to accelerate establishment of gene-disease relationships and to elucidate the genotypic and phenotypic spectrum of rare monogenic NDDs. The project received NIH funding beginning in May 2020. Participant recruitment/enrollment is ongoing across 12 Eunice Kennedy Shriver Intellectual and Developmental Disability Research Centers (IDDRCs), which form a collaborative network of institutions devoted to research into causes and treatments of NDDs [11]. Participant-level data are assembled from electronic health records (EHRs), a battery of remotely administered standardized assessments collectively referred to as the Rapid Neurobehavioral Assessment Protocol (RNAP), and co-enrollment data from the Clinical Genome Resource’s (ClinGen) GenomeConnect registry, which includes participant/caregiver completed surveys and structured genomic data collection and sharing. The purpose of this report is to provide an overview and descriptive analysis of participant enrollment from the first three years of the BGR, including distribution of genes, variant classifications, and clinical phenotypes, in order to facilitate the use of this resource by additional investigators.

## Methods

### BGR components

**Fig. 1:**
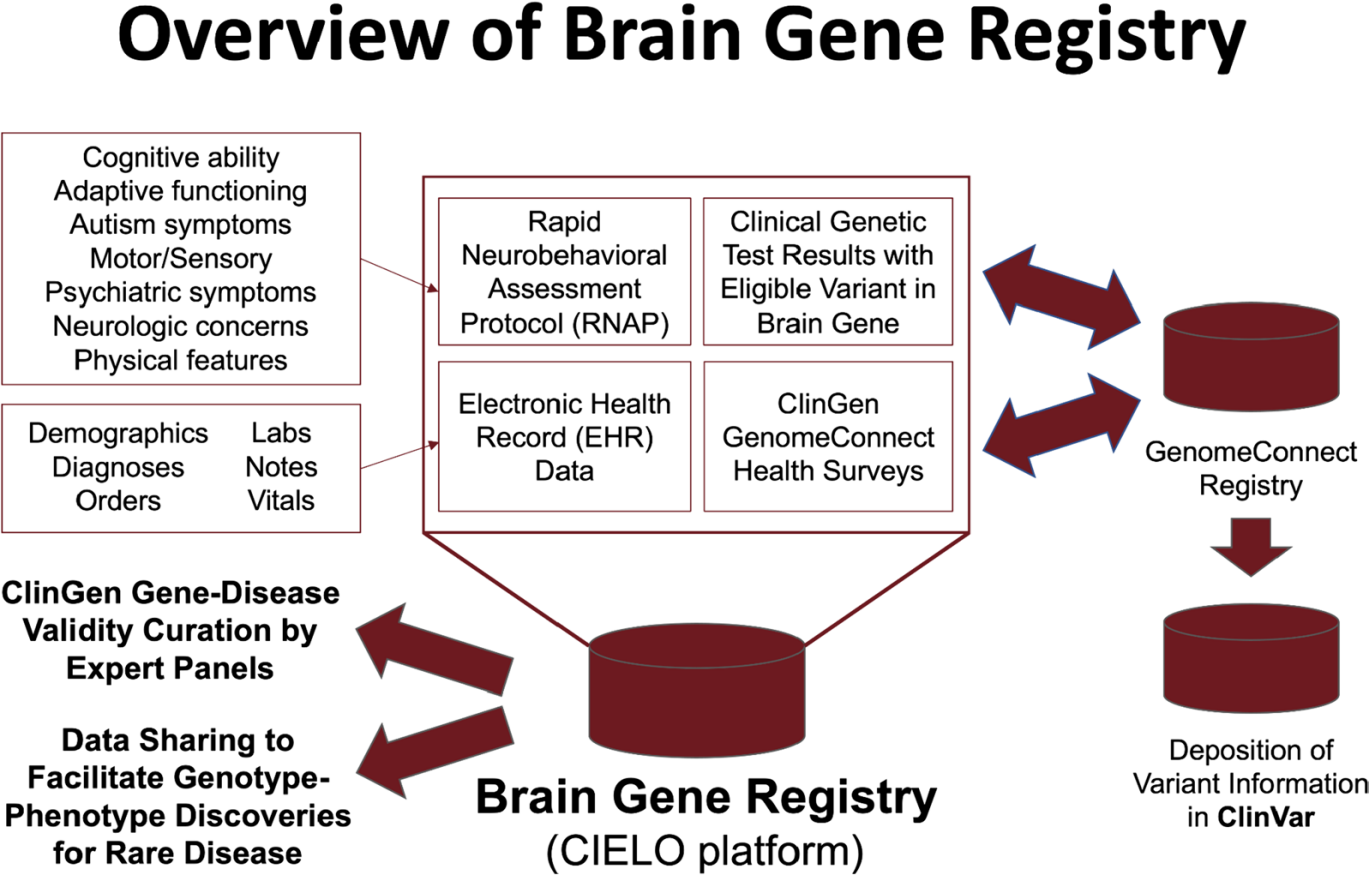
Description of Brain Gene Registry (BGR). Details are provided for BGR data elements, the relationship with GenomeConnect and ClinVar, and project outputs, including gene-disease curation and data sharing

The data collected by the BGR include EHRs, neurobehavioral assessments and surveys, clinical genetic reports, and prior records, photos, and videos (Fig. 1). In addition, BGR participants also enroll in ClinGen’s GenomeConnect [12–14] which provides participant/caregiver (for simplicity, hereafter referred to as self-reported) completed health survey information for use by the BGR, enables collection and sharing of structured genomic data with NCBI’s ClinVar, and facilitates participant recontact with variant classification updates.

Descriptive, statistical, and visual analysis of the aggregated data was performed to investigate the demographics and characteristics of the registry’s consented participants. Subject matter experts, including clinicians and geneticists, reviewed the registry data for quality and completeness given the multi-source and wide range of data points collected for the patients. Data are included from the inception of the BGR in 2020 through September 2023. Below, we provide details about the different data sources, types, formats, and analyses performed on the BGR data.

### Data commons platform

The BGR data are stored in a data commons platform named the Collaborative Informatics Environment for Learning on Health Outcomes or “CIELO,” which is a cloud-based, protected health information (PHI) secure platform that supports multiple independent research projects [15]. Data are accrued by the BGR consortium from multiple sources (EHR, RNAP, GenomeConnect) and aggregated within CIELO. Participant profiles are created that are entity records within CIELO, including basic metadata about participants, such as the recruiting institution, participant name, and sex. The profile also includes a Global Unique Identifier or GUID [16] to enable detection of overlap of participants among research projects and facilitate potential future linking of data from the same participants across collections. Subsequently, all GenomeConnect, RNAP, and EHR data are then migrated into CIELO using a bulk upload functionality which assigns individual participants’ data to their respective profiles. The final product is a sharable, unified data bundle of all information for a participant from different data sources.

### Electronic health records

EHR data was extracted from the EHR vendor in use at each collaborating institution. These data include structured data including vitals, medication orders, procedures, imaging, labs, encounters, diagnoses, and demographics, as well as unstructured data such as clinical notes. All IDDRC sites utilized the same standardized query to extract data from EHR, ensuring consistency in format and structure. Data was then uploaded to the CIELO data commons as comma-separated (CSV) files. For this study, we performed descriptive and longitudinal analyses of selected EHR data tables, such as demographics, diagnoses, encounters, and medications, including an assessment of the presence of diagnoses at each age of life to assess relative data capture and completeness. Phenome-wide association codes called Phecodes were utilized to create a consistent, concept-level roll up of ICD-10 codes from the diagnoses table of the EHR [17, 18], and were analyzed in comparison to participant age at diagnosis. Some individuals did not have available encounter or diagnoses data, leading to slight differences in the total number of participants included in each analysis.

### Rapid Neurobehavioral Assessment Protocol (RNAP)

The BGR developed and implemented a standardized neurobehavioral characterization, named the “RNAP” for Rapid Neurobehavioral Assessment Protocol [19]. The protocol consists of two major components: 1) direct assessment and observations collected via a brief telehealth visit, and 2) a selection of surveys and questionnaires completed by adult participants, or parents/caregivers of minors or adults unable to consent for themselves. The RNAP was designed to capture data from multiple domains pertinent to NDD, including cognitive ability/development, adaptive functioning, autistic symptoms, motor/sensory domain, psychiatric symptoms, neurologic concerns, and dysmorphology. The protocol was developed to be flexible across a broad age range, adaptable for remote assessment, and to consist of existing, normed instruments. The measures included gold-standard assessments such as the Shipley or DP-4, SRS-2, ASEBA-CBCL, Vineland-3, as well as disorder specific questionnaires (e.g., ADHD and autism). In the cognitive domain, only the nonverbal subtest of the Shipley was administered to accommodate potential nonverbal participants, and examiners could substitute with the DP-4 (parent/caregiver report) if the participant was too young or unable to complete the Shipley subtest. Motor scores from the Vineland-3 were only available for individuals age 9 or below.

This information was collected and managed using REDCap electronic data capture tools hosted at Washington University in St. Louis [20, 21]. REDCap (Research Electronic Data Capture) is a secure, web-based software platform designed to support data capture for research studies. The data were then exported from REDCap and uploaded to CIELO. These data were used to describe the clinical and behavioral phenotype, including cognitive ability, adaptive behavior, behavioral and emotional problems, and clinical conditions. The frequency and type of seizures associated with the genes of interest were extracted from the RNAP neurology screen.

### GenomeConnect

Variant details and participant-provided health information were obtained from GenomeConnect, the ClinGen patient registry [12, 13]. Variant information was extracted by trained GenomeConnect staff from clinical genetic testing reports uploaded by the participants. These data included variant details, report date, reporting institution, and variant classification. General health and developmental history were extracted from online surveys completed by participants/caregivers. The initial health survey captures health data across seventeen body systems, and additional surveys were assigned based on initial responses, including developmental and seizure specific surveys. Responses were uploaded to CIELO as structured data files in CSV format. GenomeConnect information was used for analyses of variant ACMG classification and variant protein effect, as well as the self-reported seizure subtypes. The frequency and type of seizure data complemented the RNAP neurology screen described above.

### Additional data

Prior records from participants were also collected, including previous neuropsychological reports, school records such as Individualized Educational Plan (IEP) evaluations, and other clinical genetic reports that were uploaded to CIELO as scanned documents, typically in PDF format. Photos of participants’ faces, hands, feet, and full-length body were collected for dysmorphology analysis and are available in CIELO. The number of participants who have records available in CIELO were used to calculate the completeness of this component of the registry data.

## Results

### Collaborating sites and participant identification

The Brain Gene Registry is a collaborative project that includes investigators across twelve IDDRCs located at major academic medical centers: Albert Einstein College of Medicine, Baylor College of Medicine, Boston Children’s Hospital, Children’s Hospital of Philadelphia, Children’s National Medical Center, Kennedy Krieger, University of California, Davis, University of North Carolina at Chapel Hill, University of Washington, Vanderbilt University Medical Center, Waisman Center, and Washington University in St. Louis. Each of these institutions contributed to the identification and enrollment of eligible research participants. Participants are identified using a variety of methods including recruitment directly from clinic visits, search of existing research databases, use of EHR queries that identify character strings corresponding to the names of particular genes of interest, and direct referrals to the BGR from patients and family advocacy groups [19].

Eligibility for enrollment in the BGR requires a clinical sequencing report documenting a pathogenic, likely pathogenic, or variant of uncertain significance in a “brain gene,” which is defined as a gene with at least nominal evidence of association with NDD phenotype(s) or “implicated in neurodevelopment with varying degrees of evidence” [19]. Enrollment was initially deprioritized for individuals with variants in multiple brain genes in order to enable clear assertions of gene-disease relationships, but has now expanded to include participants with multiple variants. Genes of interest are periodically reviewed, and the current gene list is included on the BGR website (https://braingeneregistry.wustl.edu).

### Demographics and data completeness

**Fig. 2:**
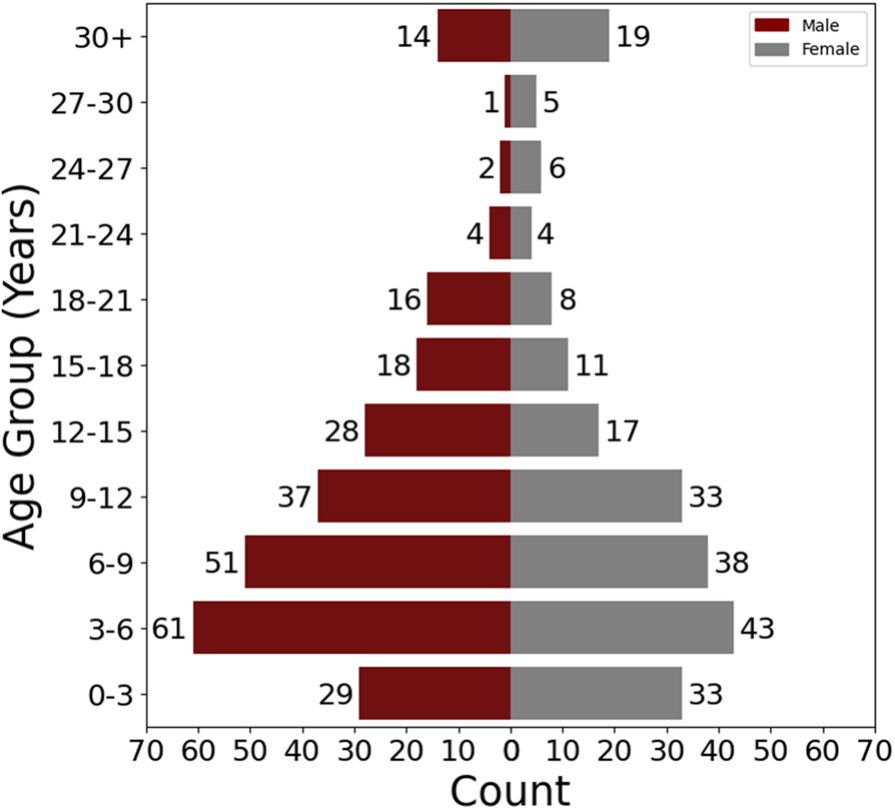
Age and sex distribution of enrolled BGR Participants (N = 479). Male (maroon bars) and female (gray bars) BGR participants are binned into 3-year age groups

As of September 2023, 479 participants were enrolled in the BGR (Table 1, Fig. 2), which includes 262 males and 217 females. Participants had an average age of 11.8 years (SD = 10). The self-reported race of the participants was 6% Asian, 6% Black or African American, and 76% white. Hispanic or Latine ethnicity was reported by 12% of participants. Data completeness, defined as number of participants for whom each data type was obtained, was variable, with RNAP data collected from 298 individuals (62%), creation of a unique user ID (GUID) and data commons participant profile for 388 individuals (81%), GenomeConnect data for 241 individuals (50%), and EHR data for 218 individuals (46%) (Supplementary Fig. 1). Data completeness by sex, race, and ethnicity were similar to the overall cohort (See Supplementary Table 1, Additional File 1). Dysmorphology assessments and photos are available for 298 (62%) and 32 (7%) individuals, respectively.

**Table 1:** Demographics of BGR cohort participants, including self-reported sex, race, and ethnicity

Sex	Female	217 (45%)
	Male	262 (55%)
Ethnicity	Hispanic or Latine	58 (12%)
	Not Hispanic or Latine	390 (81%)
	Unknown / Not Reported	31 (7%)
Race	Asian	27 (6%)
	Black or African American	30 (6%)
	More Than One Race	25 (5%)
	Other	19 (4%)
	Unknown / Not Reported	13 (3%)
	White	363 (76%)

### Clinically reported gene variants

**Fig. 3:**
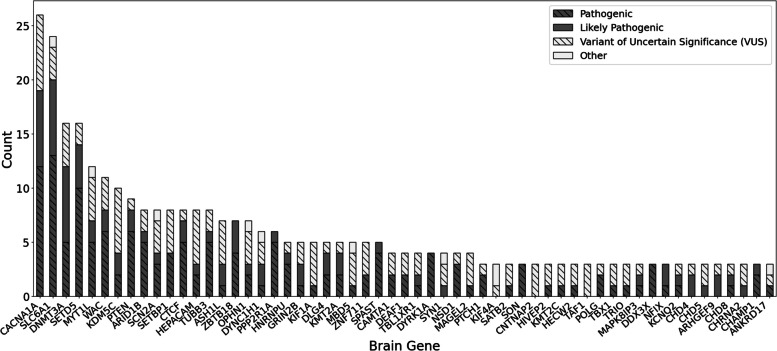
Classification of the most common brain gene variants within the BGR**.** Only genes in which there are variants in 3 or more individuals are shown. 50 individuals have variants in more than one brain gene and are represented multiple times in this analysis

In 479 total participants, variants in 241 unique genes were reported (Additional File 2). Genes with the most reported variants were *CACNA1A* (n = 26), *SCL6A1* (n = 24), *DNMT3A* (n = 16), and *SETD5* (n = 16) (Fig. 3). In total, 30% of variants are de novo (116/391) and 43% (242/564) are variants of uncertain significance (VUSs) (Supplementary Figs. 2–4). Most participants have a single clinically relevant sequence variant identified (Supplementary Fig. 5). Data from the BGR has been considered in the curation of 36 gene-disease relationships by the ID/autism ClinGen GCEP, which includes members of the BGR team.

### Neurobehavioral assessments

**Fig. 4:**
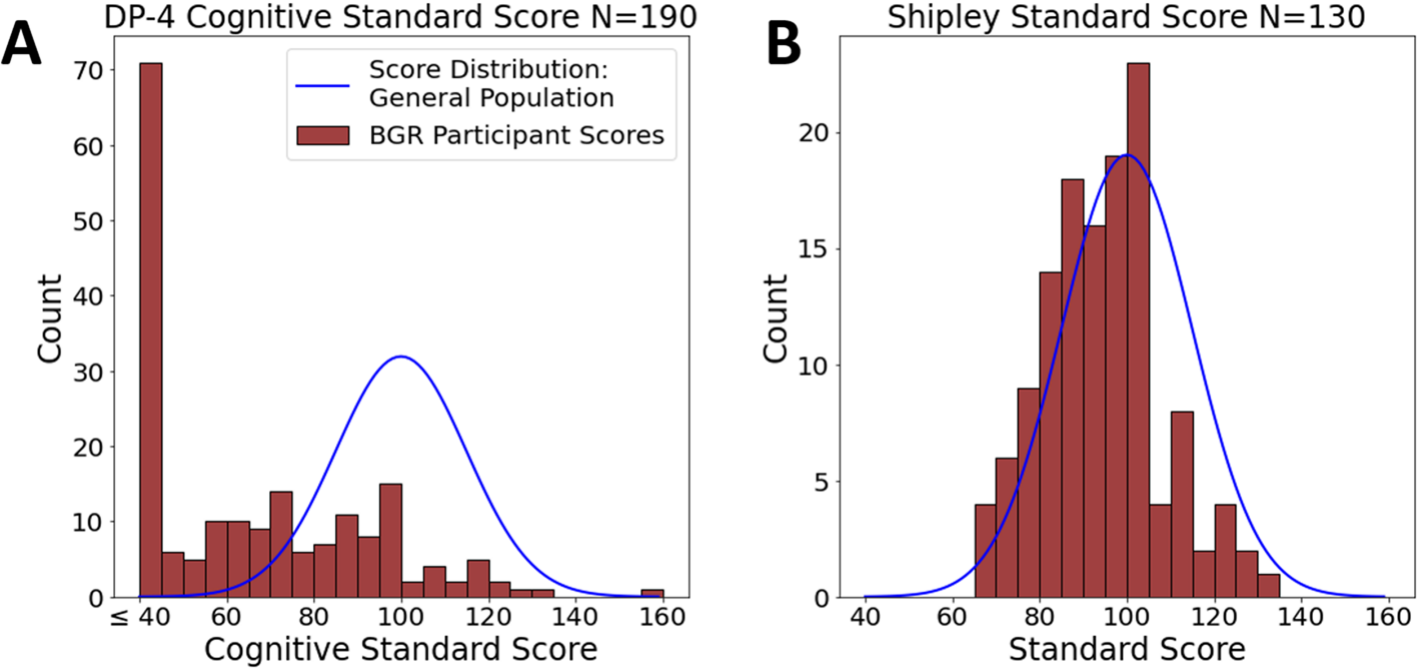
Distribution of cognitive scores for BGR participants. **A**) DP-4 assessment (*N* = 190), **B**) Shipley assessment (*N* = 130). Scores of < 40 (4 SD below General Population) were recorded as 40 for the DP-4

**Fig. 5:**
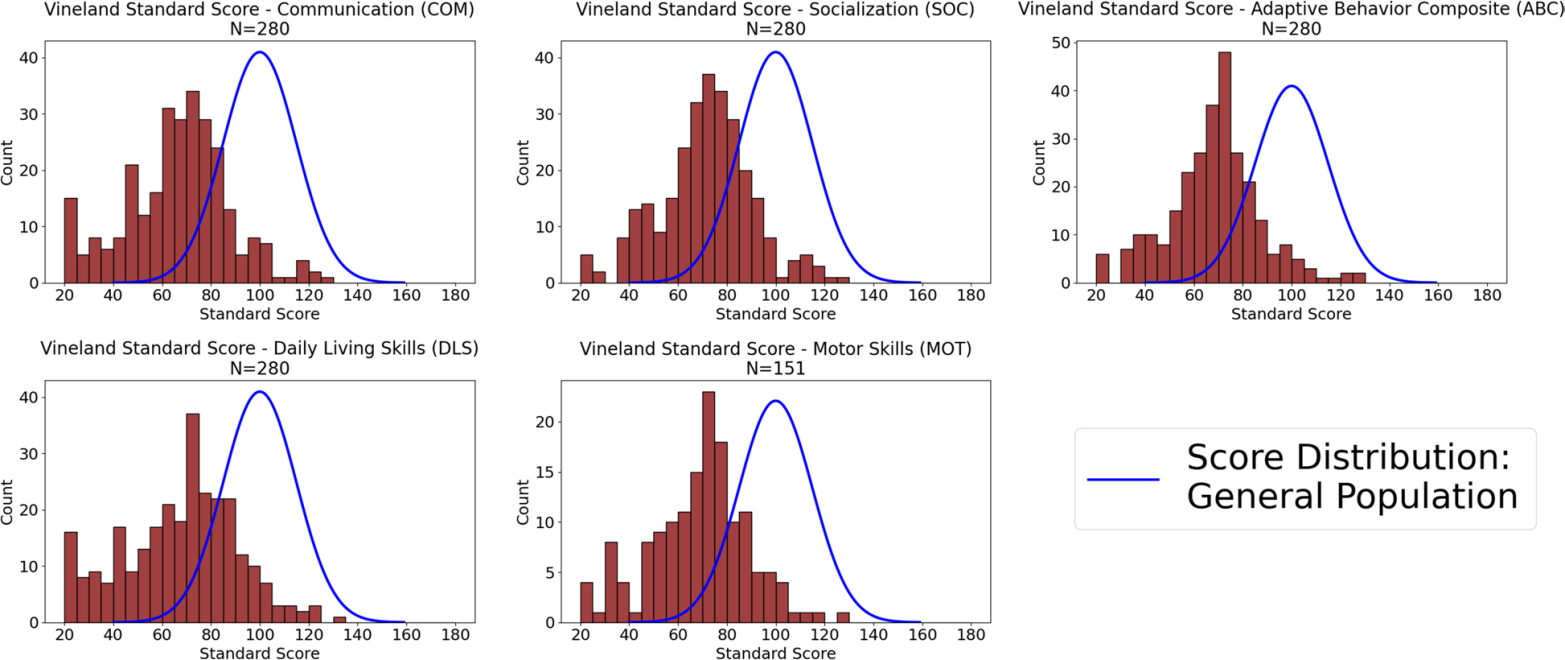
Distribution of Vineland-3 standard scores across 5 behavior categories. Distribution of scores observed in the entire BGR cohort (*N* = 479). Blue line indicates the general population score distribution

**Fig. 6:**
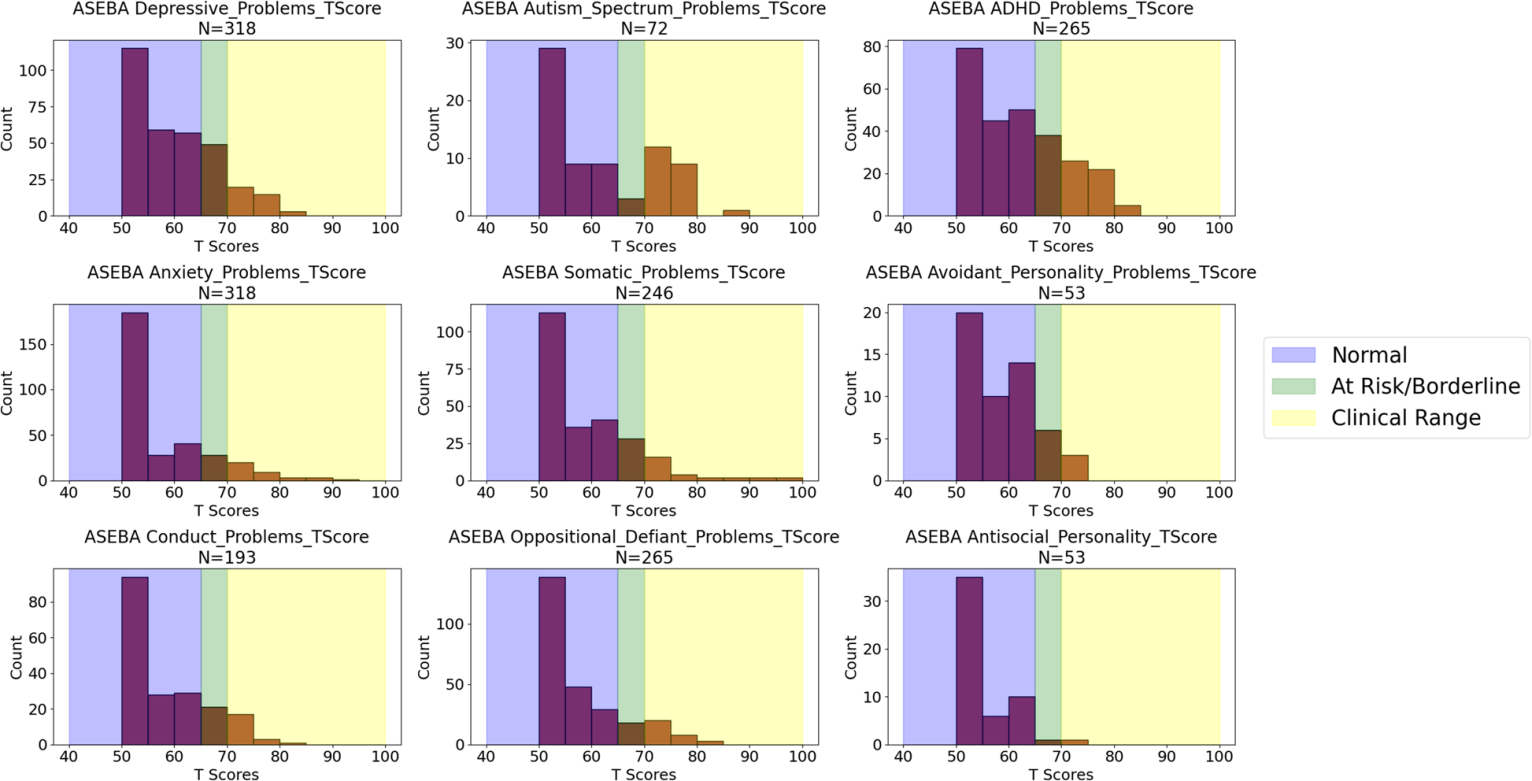
Distribution of ASEBA-CBCL behavior report T-Scores for individuals within the registry. Score areas are color-coded indicating normal scores (< 65, blue), at-risk or borderline scores (65–70, green), and clinical range scores (> 70, yellow)

Results of the RNAP assessment, which were completed virtually via telehealth visits, were available on 298 participants (62% of the cohort). To assess nonverbal cognitive ability, the Shipley Block Patterns subtest was administered to participants able to actively participate in a video interview with the examiner, while the DP-4 (cognitive subscale) parent/caregiver checklist was utilized if participants were either too young or too severely affected to complete the Shipley. Cognitive testing results demonstrated that 3.1% of participants scored below SS = 70 (intellectually impaired range) on the Shipley, while 58.4% scored below 70 on the DP-4 (mean = 65.82, standard deviation = 25.98) (Fig. 4). Lower scores on the DP-4 were expected, as the DP-4 was utilized for more severely affected individuals. Adaptive behavior assessment (Vineland-3) indicated overall scores (ABC scores) occurred with a bell curve score distribution that was shifted below the general population (mean = 67.92) (Fig. 5). Report of problem behaviors (ASEBA-CBCL) across the cohort showed parent/caregiver concerns in all areas, with more concerns noted for ADHD (34.3% in the at-risk range) and autism-related (34.7% in the clinical range) symptoms (Fig. 6).

### Description of electronic health record (EHR) data

**Fig. 7:**
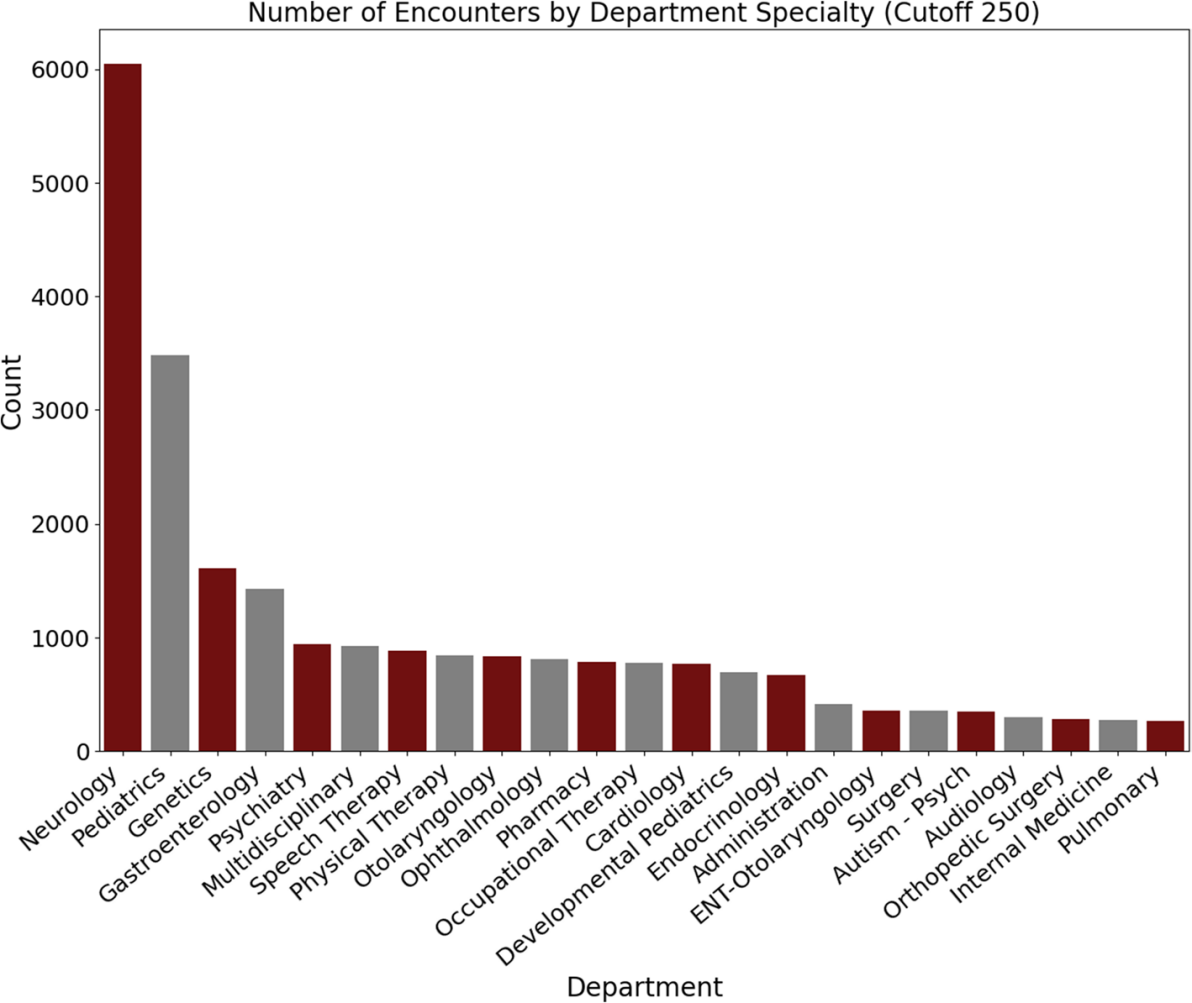
Medical specialty encounters in the BGR. Data were obtained from available electronic health records with encounter data (N = 179 participants)

For BGR participants for whom EHR encounter records were available (*N* = 198, 41% of cohort), the number of clinical encounters in each clinical specialty documented in their EHR was determined, along with corresponding baseline data characteristics (Supplementary Figs. 6–9). The specialty with the most visits was neurology, followed by pediatrics, and genetics (Fig. 7). We also observed that our participants had frequent visits for speech, physical, and occupational therapy.

**Fig. 8:**
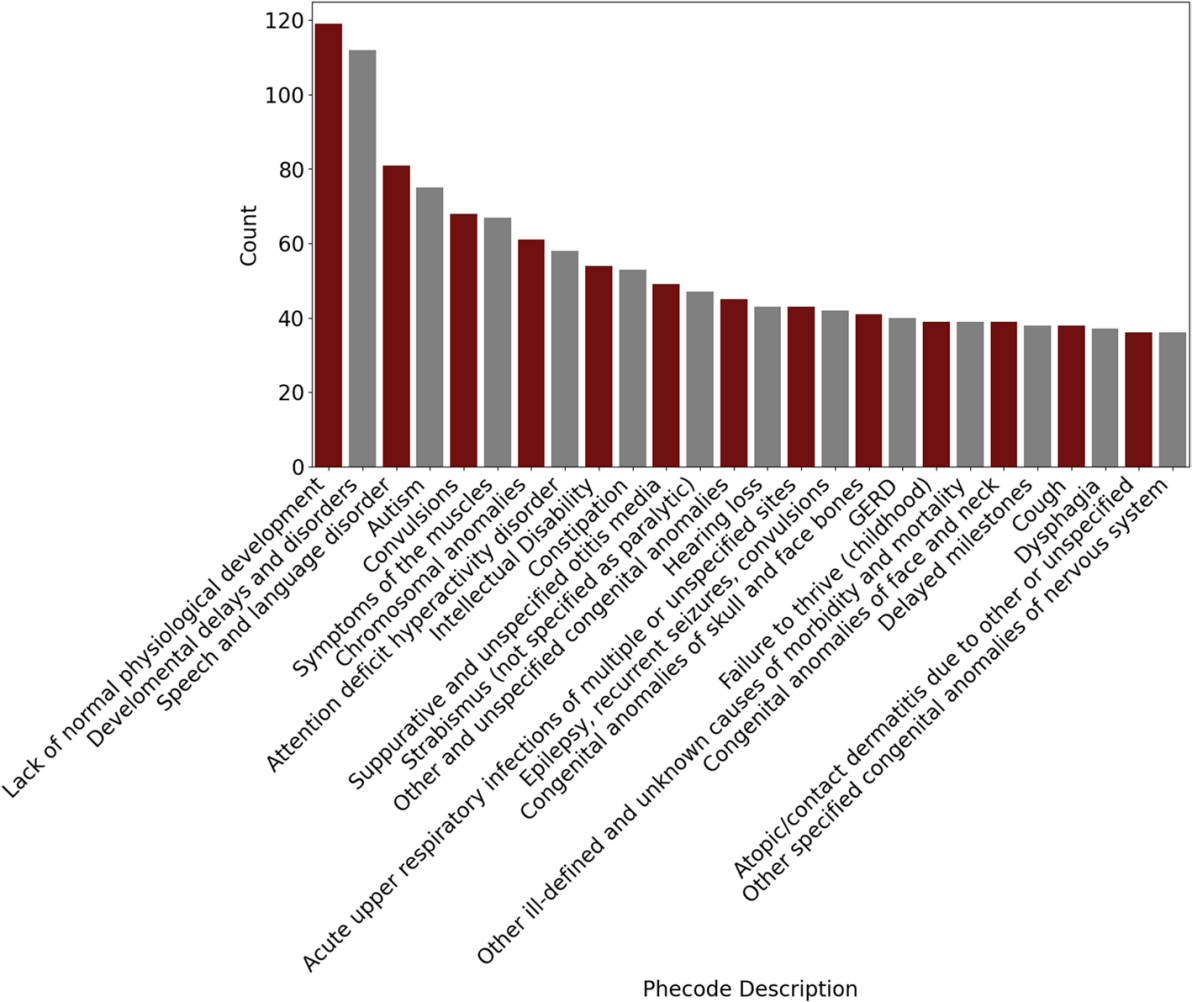
Phecode diagnoses across the BGR cohort. Phecode data were derived from BGR participants for whom ICD-10 codes were available in their electronic health records in CIELO (N = 207). Individual participants are represented only once per Phecode

**Fig. 9:**
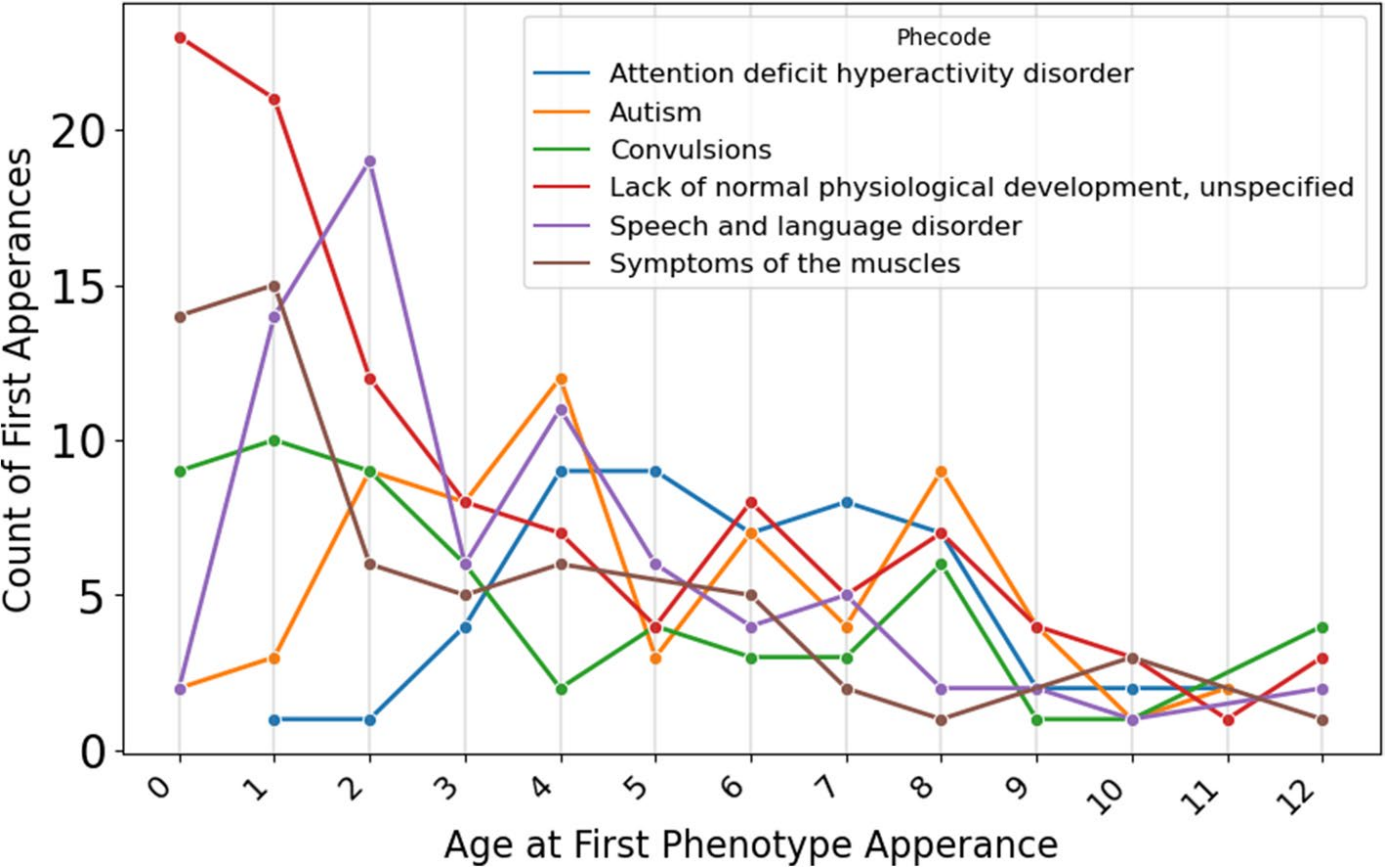
Distribution of BGR participant age at the first appearance of each Phecode (N = 207 patients). The number of participants with an ICD-10 code corresponding to the specified Phecode is plotted according to the participant’s age at first appearance of that Phecode in their medical record. A single participant is represented only once in a given Phecode

To determine the most frequent category of diagnoses present in the BGR cohort, ICD diagnoses present in the EHR of BGR participants were grouped using Phecodes (207 participants, 43% of cohort). Phecodes are a manual grouping of similar ICD-10 codes used to ease comparison of the most common diagnoses present in the cohort [17, 18]. Overall, the most commonly observed Phecodes included abnormal development, developmental disorders, autism, and seizures, which are noted as “convulsions” in the Phecode nomenclature (Fig. 8). The age at which the participant’s diagnosis first appeared in their EHR records was used as a proxy for the age of diagnosis, although we recognize that other factors may influence when a diagnosis appears in a participant’s medical record, such as when the individual first received care in that healthcare system, and when the particular institution began using that medical record system. Developmental disorders appeared earliest in the participants’ EHRs, being most prevalent between birth and one year of age (Fig. 9). Diagnosis of speech and language disorder peaks at 2 years, and autism peaks at 4 years. Among these conditions, the overall median time between first Phecode appearance and age of enrollment was 3.9 years (Supplementary Fig. 10).

### Use case of seizure data

**Fig. 10:**
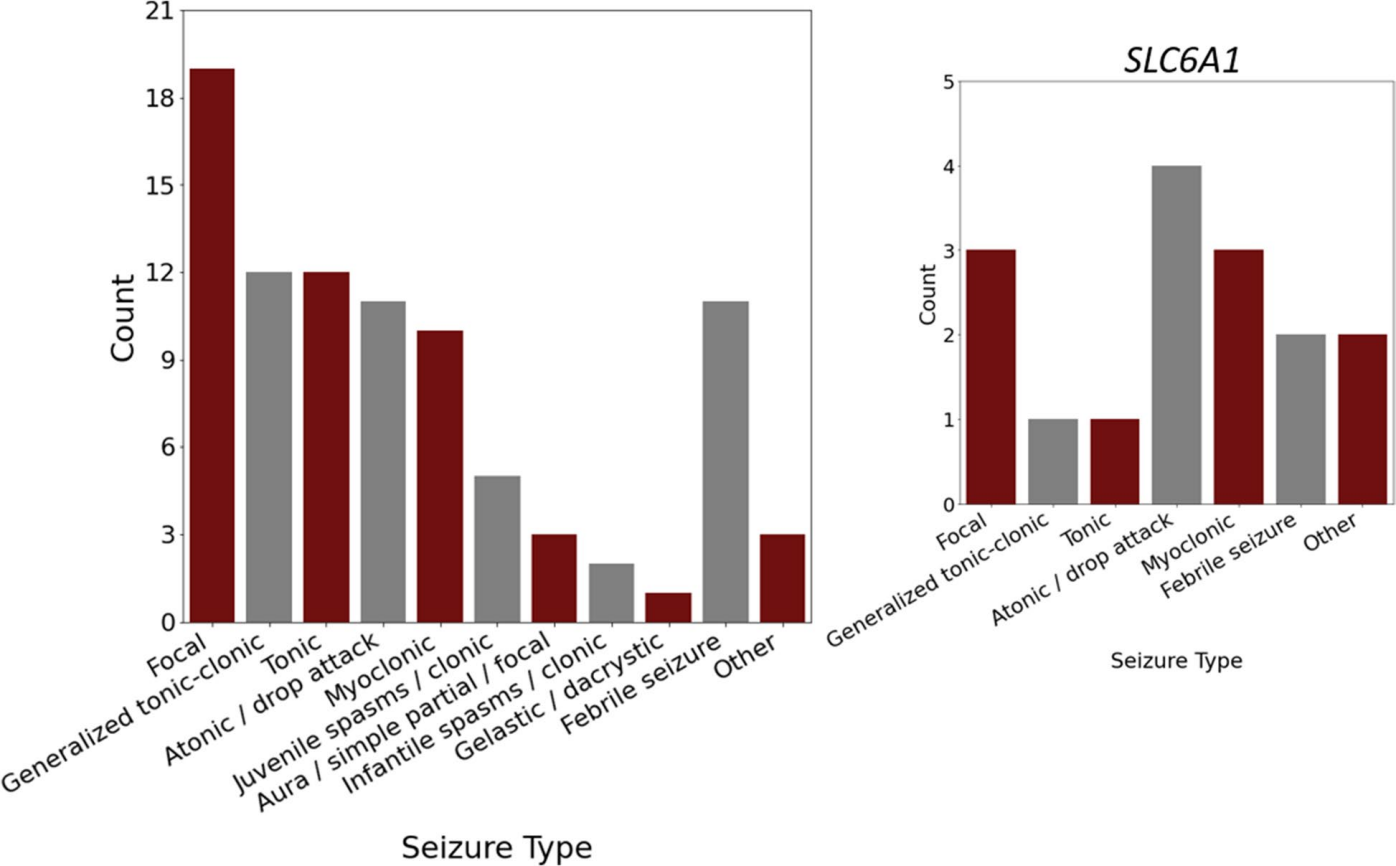
Frequency of self-reported seizure types among all BGR participants (*N* = 34) (left) compared to participants with *SLC6A1* variants (*N* = 5) (right). Data was generated from the participant/parent self-report GenomeConnect seizure survey. A single participant may report multiple different types of seizures in the same survey, leading to higher total count than the number of participants. The focal seizure data points include those with atypical absence and complex partial seizures. Generalized tonic–clonic includes grand mal seizures and general convulsions. Individuals with *SLC6A1* variants are shown because it was the most common gene among participants who took the survey

To explore the richness and potential overlap of the data collected on BGR participants and to provide an example use case leveraging the GenomeConnect health surveys and the RNAP, we evaluated the data regarding participant seizures. Seizures were self-reported via the GenomeConnect health survey in 23.2% (56/241) of participants. Among the 34 participants reporting seizures who also completed the self-reported GenomeConnect seizure type survey, focal seizures were the most common, followed by generalized tonic–clonic, and tonic seizures (Fig. 10). When subsetting the results for the five participants with variants in *SLC6A1*, atonic/drop attack, focal, and myoclonic seizures were the most common. We excluded 22 additional participants who indicated the presence of seizures in a GenomeConnect general health survey, but seizure type was not indicated.

**Fig. 11:**
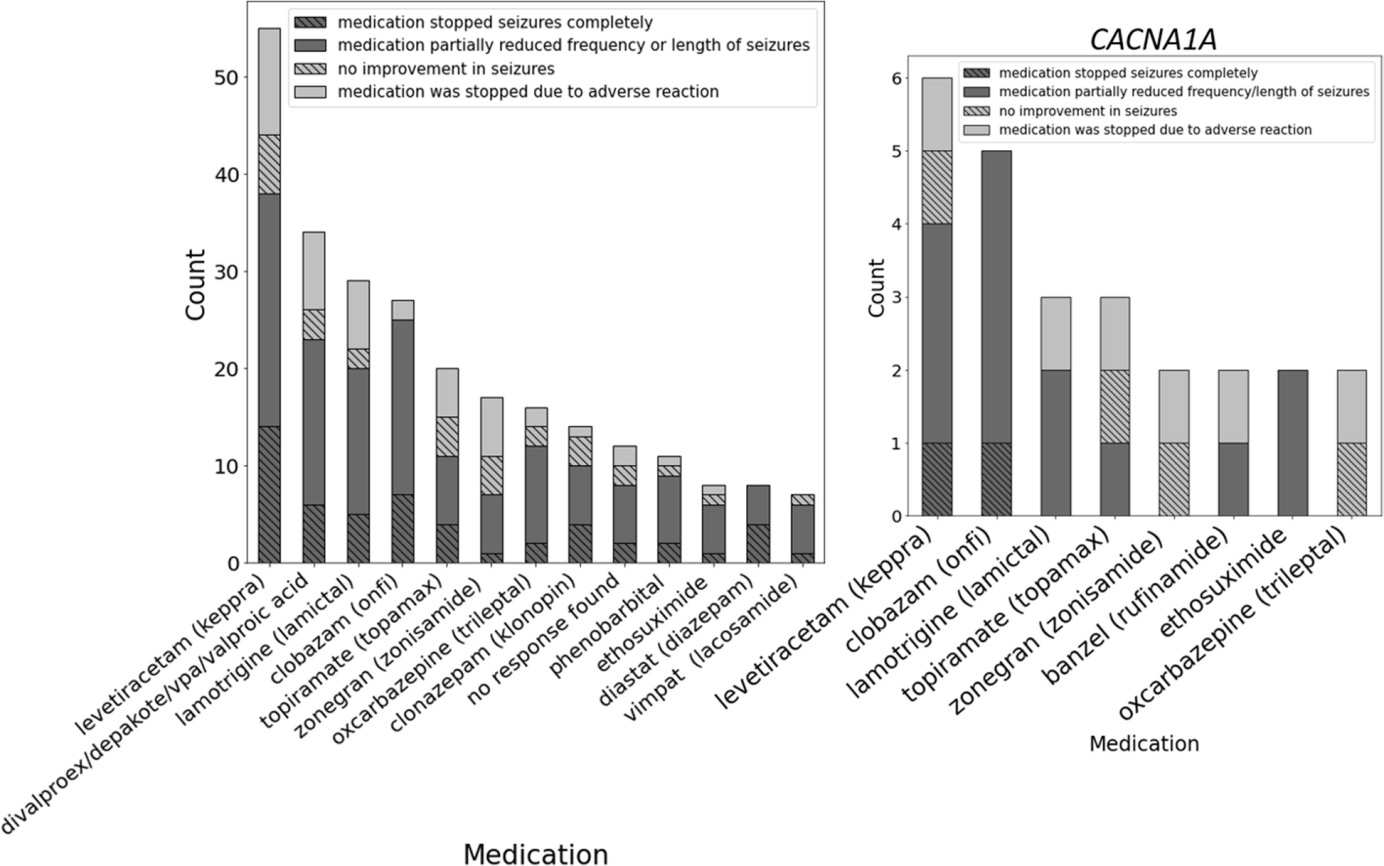
Seizure medication use among all BGR participants (*N* = 110) (left) compared to participants with *CACNA1A* variants (*N* = 7) (right). Data represent participants who completed the self-reported seizure survey in the RNAP. A single individual may report multiple seizure medications. Individuals with a *CACNA1A* variant are shown because it was the most gene among participants who completed the survey

Of the participants who completed the seizure medication survey as part of the RNAP, levetiracetam was the most common seizure medication, followed by valproic acid and lamotrigine (Fig. 11, Supplementary Fig. 11). For participants with *CACNA1A* variants, which was the most represented gene in the survey, levetiracetam was the most common medication, followed by clobazam and lamotrigine. Participants also reported the effect of the medications on their seizures. For the cohort overall, levetiracetam was self-reported to have had the greatest impact on reducing or eliminating seizures. For the *CACNA1A* subcohort, levetiracetam and clobazam both were reported as efficacious, while zonegran and oxcarbazepine were reported to have had no effect or caused an adverse reaction.

**Fig. 12:**
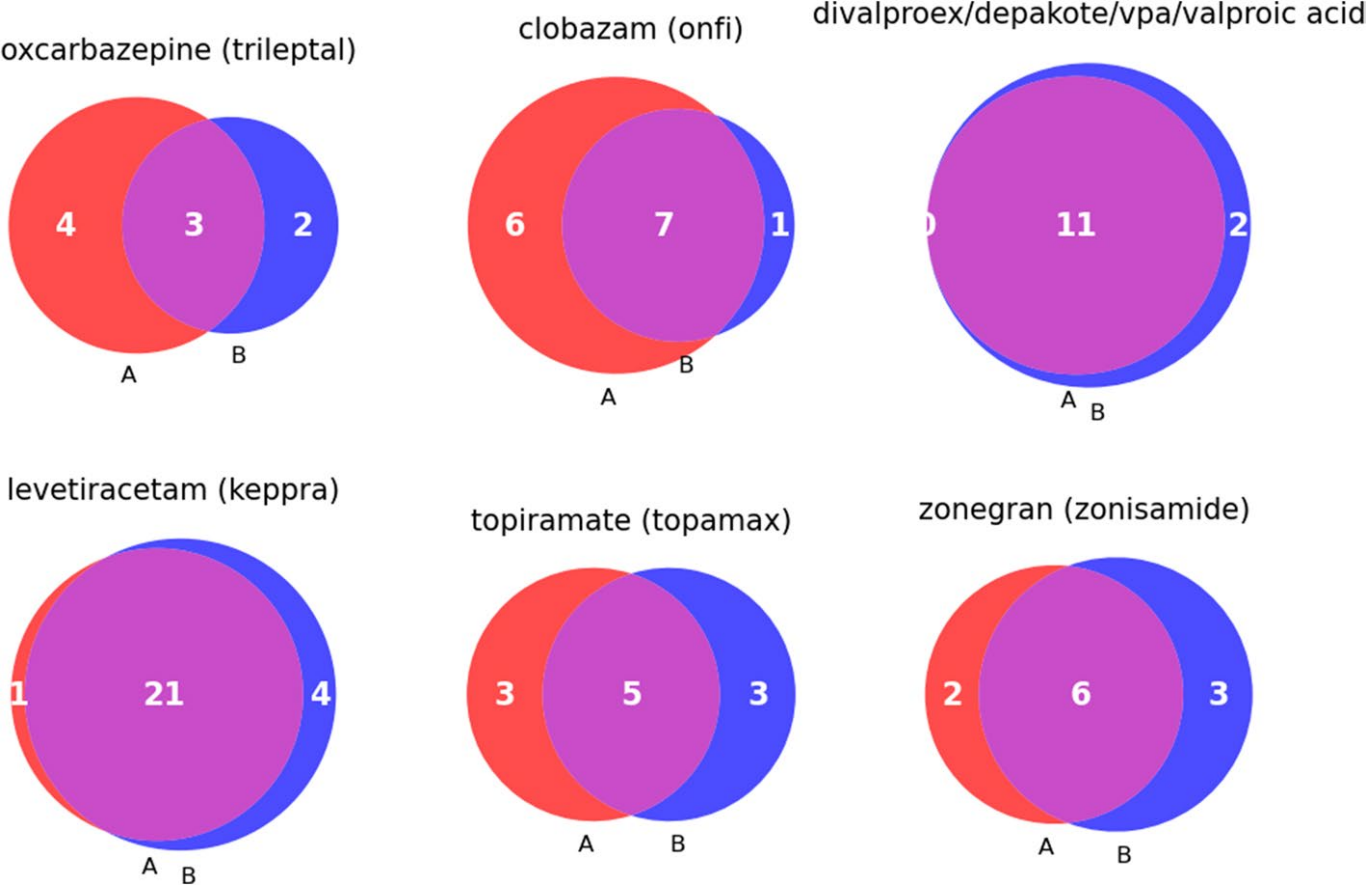
Venn diagrams showing the overlap of seizure medication usage among BGR participants comparing self-reported data with EHR data. Only participants with both the RNAP seizure survey data and EHR extracted medication data are included (*N*=38).

Of the participants who completed the RNAP seizure survey (*N* = 110), there were 38 who also had EHR-derived medication order history enabling comparison between self-reported medication and EHR data. Participants in which both data were collected were used to evaluate concordance across self-report and EHR data for seizure-related medications (Fig. 12). Valproic acid and levetiracetam had the greatest overlap of the number of patients reporting the same medication usage in the RNAP and EHR order history. The lack of concordance may be due to multiple factors, including the use of these medications for conditions other than seizures, incomplete EHR data, orders being unequal to medication fills and administration, poor recall of medications prescribed, or prescription prior to EHR implementation.

## Discussion

The Brain Gene Registry (BGR) is a central IDDRC resource that is designed to collect genotypic and phenotypic information from participants for maximal usage by a variety of stakeholders. The content and format of the data collected by the BGR is designed with end-users in mind, with a goal of increasing the number of individuals (investigators, patient advocacy groups, gene, and variant curation panels) who use these data in their own research. The BGR prioritizes recruitment of participants with variants in genes nominated by investigators and genes present on commercially tested ID/autism panels, leading to larger numbers of participants with variants in specific genes. Further, specific collaborations that the BGR has established with clinicians, researchers, and patient advocacy groups resulted in increased enrollment for some genes, including *CACNA1A, SLC6A1,* and *DNMT3A*. The aggregation of additional cases of participants with variants in genes of interest coupled with the deep phenotypic data contained within the BGR makes the data valuable for curation efforts [19]. A future vision for the BGR includes the addition of a dedicated BGR Variant Curation Expert Panel (VCEP) as part of the larger ClinGen curation effort. This panel would review genetic and phenotypic data from the published literature and from the BGR database to provide consensus classifications, including reevaluation of VUS. We also anticipate future systematic analyses of VUS present in the BGR, including potential phenotype expansions, as well as inheritance and race/ethnicity considerations.

The utility of EHR data for rare disease research has been poorly studied due to the challenges of accumulating data across multiple institutions [22]. The IDDRC network is a major asset for rare disease research by supporting the BGR through local referral and recruitment of participants across its many sites who might otherwise not be identified for research. Furthermore, the collection of EHR data across multiple sites also makes the BGR an ideal resource to compare data standardization and content across institutions. Data redundancy within the compiled BGR dataset can also be studied, as we have done here with seizure medication data, in order to streamline future data collection and reduce participant burden. We have future plans to evaluate the effectiveness of artificial intelligence based predictive phenotyping models and algorithmic extraction of phenotypes from the EHR derived data to further reduce participant burden for rare disease research.

There are several limitations of the BGR. First, collecting complete sets of participant data from multiple sources, including the EHR and self-reported survey data, is a challenge. Participant burden is high, even though the phenotypic assessment is less than one hour of face-to-face time and completed virtually by phone or tablet, and data is often incomplete. Second, it is challenging to recruit multiple participants with variants in the same rare disease gene even when twelve centers are recruiting, particularly for ultra-rare disorders. By nature of the way in which genetic data is stored in the EHR at each IDDRC, which is typically in PDF format when returned by a clinical laboratory, the IDDRCs sites have variable capacities to identify individuals with variants in specific genes. Some institutions have existing research repositories maintained by clinical geneticists which may have influenced which types of participants were recruited. There is likely bias toward recruitment of participants with moderate to severe neurodevelopmental disability as reflected by the Phecode observations and by the high numbers of visits to neurologists in the encounters data and the frequent visits for speech, physical, and occupational therapy. This bias likely reflects the types of patients who currently receive clinical diagnostic genetic testing.

Bias in clinical genetic testing also likely explains our failure to recruit BGR participants who are fully representative of the racial and ethnic composition of the US population. For example, Black participants comprise only 6% of our cohort versus 13.6% of the US population, and Hispanic participants make up 12% of our cohort versus 19% of the US population [23]. Despite deliberate efforts to improve diversity amongst participants with the inclusion of Spanish-language consent materials and dedicated Spanish-speaking BGR team members, multiple factors, including major health disparities in genetic testing [24, 25] and language barriers contributed to the over-representation of white individuals. Lack of representation of diverse populations in genetics databases and genetics research is widespread and contributes to health disparities [26, 27]. The underrepresentation of individuals who self-reported their race as Black or African American in the Brain Gene Registry compared to the United States Census (13.6%) is likely a result of both underrepresentation of Black and African American individuals in genetic research efforts and barriers this population faces in receiving specialized care allowing for genetic testing. Both underrepresentation in genetic research and decreased access to genetic testing are a result of historic and systemic racism and medical mistreatment particularly impacting Black and African American individuals. Medical bias, mistrust of physicians and researchers, decreased referrals by primary care physicians, socioeconomic barriers, and clinical care and study designs which lack cultural competency all contribute to the underrepresentation of Black and African American individuals in genetic research, including the Brain Gene Registry [24, 28–31]. Because VUSs are more common in underrepresented populations, the BGR is focused on recruiting participants with VUSs not only to increase representation and research opportunities for underrepresented populations, but as a source of data for ClinGen curation efforts to improve gene-disease validity curation and variant classification.

The goal of the BGR is to create an IDDRC-supported resource for the use and benefit of multiple partners, including academic researchers, patient advocacy groups, and the patients themselves. BGR participants consent for future recontact, which is relevant for future clinical trials, translational research, and variant interpretation updates. To date, nine BGR participants have received a variant classification update from GenomeConnect. We are also making continuous iterative improvements to the CIELO platform and our dashboard to facilitate data sharing. The BGR encourages use of its data and follows standard data sharing processes, including regular review of requests for data access for approval. Data requests can be made at this website: https://braingeneregistry.wustl.edu/.

### Supplementary Information

**Additional File 1. **One Table and Eleven Supplementary Figures that provide additional contextual information and analyses.

**Additional File 2. **Count of Brain Gene Variants. The number of variants observed for each brain gene included in the BGR.

## Data Availability

The dataset supporting the conclusions of this article is available in the Brain Gene Registry repository, https://braingeneregistry.wustl.edu/.

## Abbreviations

BGR: Brain Gene Registry
RNAP: Rapid Neurobehavioral Assessment Protocol
EHR: Electronic Health Record
ASD: Autism spectrum disorder
ADHD: Attention deficit hyperactivity disorder
NDD: Neurodevelopmental disorder
ID: Intellectual disability
VUSs: Variants of uncertain significance

## References

[1] Association AP (2013). Diagnostic and statistical manual of mental disorders : DSM-5.

[2] Srivastava S, Love-Nichols JA, Dies KA, Ledbetter DH, Martin CL, Chung WK (2019). Meta-analysis and multidisciplinary consensus statement: exome sequencing is a first-tier clinical diagnostic test for individuals with neurodevelopmental disorders. Genet Med.

[3] Srivastava S, Lewis SA, Cohen JS, Zhang B, Aravamuthan BR, Chopra M (2022). Molecular Diagnostic Yield of Exome Sequencing and Chromosomal Microarray in Cerebral Palsy: A Systematic Review and Meta-analysis. JAMA Neurol.

[4] Manickam K, McClain MR, Demmer LA, Biswas S, Kearney HM, Malinowski J (2021). Exome and genome sequencing for pediatric patients with congenital anomalies or intellectual disability: an evidence-based clinical guideline of the American College of Medical Genetics and Genomics (ACMG). Genet Med.

[5] Baldridge D, Heeley J, Vineyard M, Manwaring L, Toler TL, Fassi E (2017). The Exome Clinic and the role of medical genetics expertise in the interpretation of exome sequencing results. Genet Med.

[6] Richards S, Aziz N, Bale S, Bick D, Das S, Gastier-Foster J (2015). Standards and guidelines for the interpretation of sequence variants: a joint consensus recommendation of the American College of Medical Genetics and Genomics and the Association for Molecular Pathology. Genet Med.

[7] van der Sanden B, Schobers G, CorominasGalbany J, Koolen DA, Sinnema M, van Reeuwijk J (2023). The performance of genome sequencing as a first-tier test for neurodevelopmental disorders. Eur J Hum Genet.

[8] Sanchis-Juan A, Megy K, Stephens J, ArmirolaRicaurte C, Dewhurst E, Low K (2023). Genome sequencing and comprehensive rare-variant analysis of 465 families with neurodevelopmental disorders. Am J Hum Genet.

[9] Gudmundsson S, Singer-Berk M, Watts NA, Phu W, Goodrich JK, Solomonson M (2022). Variant interpretation using population databases: Lessons from gnomAD. Hum Mutat.

[10] Rehm HL, Alaimo JT, Aradhya S, Bayrak-Toydemir P, Best H, Brandon R (2023). The landscape of reported VUS in multi-gene panel and genomic testing: Time for a change. Genet Med.

[11] Walkley SU, Abbeduto L, Batshaw ML, Bhattacharyya A, Bookheimer SY, Christian BT (2019). Intellectual and developmental disabilities research centers: Fifty years of scientific accomplishments. Ann Neurol.

[12] Kirkpatrick BE, Riggs ER, Azzariti DR, Miller VR, Ledbetter DH, Miller DT (2015). GenomeConnect: matchmaking between patients, clinical laboratories, and researchers to improve genomic knowledge. Hum Mutat.

[13] Savatt JM, Azzariti DR, Faucett WA, Harrison S, Hart J, Kattman B (2018). ClinGen's GenomeConnect registry enables patient-centered data sharing. Hum Mutat.

[14] Savatt JM, Azzariti DR, Ledbetter DH, Palen E, Rehm HL, Riggs ER, Martin CL (2021). Recontacting registry participants with genetic updates through GenomeConnect, the ClinGen patient registry. Genet Med.

[15] Payne P, Lele O, Johnson B, Holve E (2017). Enabling Open Science for Health Research: Collaborative Informatics Environment for Learning on Health Outcomes (CIELO). J Med Internet Res.

[16] Johnson SB, Whitney G, McAuliffe M, Wang H, McCreedy E, Rozenblit L, Evans CC (2010). Using global unique identifiers to link autism collections. J Am Med Inform Assoc.

[17] Denny JC, Bastarache L, Ritchie MD, Carroll RJ, Zink R, Mosley JD (2013). Systematic comparison of phenome-wide association study of electronic medical record data and genome-wide association study data. Nat Biotechnol.

[18] Denny JC, Ritchie MD, Basford MA, Pulley JM, Bastarache L, Brown-Gentry K (2010). PheWAS: demonstrating the feasibility of a phenome-wide scan to discover gene-disease associations. Bioinformatics.

[19] Chopra M, Savatt JM, Bingaman TI, Good ME, Morgan A, Cooney C (2023). Clinical variants paired with phenotype: A rich resource for brain gene curation. Genet Med.

[20] Harris PA, Taylor R, Minor BL, Elliott V, Fernandez M, O'Neal L (2019). The REDCap consortium: Building an international community of software platform partners. J Biomed Inform.

[21] Harris PA, Taylor R, Thielke R, Payne J, Gonzalez N, Conde JG (2009). Research electronic data capture (REDCap)–a metadata-driven methodology and workflow process for providing translational research informatics support. J Biomed Inform.

[22] Lewis AE, Weiskopf N, Abrams ZB, Foraker R, Lai AM, Payne PRO, Gupta A (2023). Electronic health record data quality assessment and tools: a systematic review. J Am Med Inform Assoc.

[23] Bureau USC. QuickFacts United States n.d. Available from: https://www.census.gov/quickfacts/fact/table/US/PST045222. Accessed 26 Feb 2024.

[24] Frazier ZJ, Brown E, Rockowitz S, Lee T, Zhang B, Sveden A (2023). Toward representative genomic research: the children's rare disease cohorts experience. Ther Adv Rare Dis.

[25] Odgis JA, Gallagher KM, Suckiel SA, Donohue KE, Ramos MA, Kelly NR (2021). The NYCKidSeq project: study protocol for a randomized controlled trial incorporating genomics into the clinical care of diverse New York City children. Trials.

[26] Petrovski S, Goldstein DB (2016). Unequal representation of genetic variation across ancestry groups creates healthcare inequality in the application of precision medicine. Genome Biol.

[27] Hindorff LA, Bonham VL, Brody LC, Ginoza MEC, Hutter CM, Manolio TA, Green ED (2018). Prioritizing diversity in human genomics research. Nat Rev Genet.

[28] Chapman-Davis E, Zhou ZN, Fields JC, Frey MK, Jordan B, Sapra KJ (2021). Racial and Ethnic Disparities in Genetic Testing at a Hereditary Breast and Ovarian Cancer Center. J Gen Intern Med.

[29] Brown RF, Cadet DL, Houlihan RH, Thomson MD, Pratt EC, Sullivan A, Siminoff LA (2013). Perceptions of participation in a phase I, II, or III clinical trial among African American patients with cancer: what do refusers say?. J Oncol Pract.

[30] Walley NM, Pena LDM, Hooper SR, Cope H, Jiang YH, McConkie-Rosell A (2018). Characteristics of undiagnosed diseases network applicants: implications for referring providers. BMC Health Serv Res.

[31] Fraiman YS, Wojcik MH (2021). The influence of social determinants of health on the genetic diagnostic odyssey: who remains undiagnosed, why, and to what effect?. Pediatr Res.

