# Supplementary material for: The Brain Gene Registry: a data snapshot
Source: J Neurodev Disord. 2024 Apr 17;16:17. doi: 10.1186/s11689-024-09530-3 (PMC11022437; doi:10.1186/s11689-024-09530-3)
Supplement: Supplementary file 1 — Additional File 1. One Table and Eleven Supplementary Figures that provide additional contextual information and analyses. [file 11689_2024_9530_MOESM1_ESM.docx]

**Additional File 1.**

**Supplementary Table 1 and Figures for the manuscript, The Brain Gene Registry: A Data Snapshot**

**Supplementary Table 1**. Demographic characteristics of BGR cohort and counts of data types available.

| **Measurement** | **Category** | **Enrolled and e-Consented** | **RNAP Data Collection Complete** | **Data Commons Profile Created** | **GenomeConnect Data Extracted** | **EHR Data Extracted** |
| --- | --- | --- | --- | --- | --- | --- |
| **N** |  | 479 | 298 | 388 | 241 | 218 |
| **Age (SD)** |  | 11.8 (10.0) | 12.2 (9.9) | 11.3 (9.2) | 11.1 (8.8) | 10.9 (8.1) |
| **Ethnicity** | **Hispanic or Latine** | 58 (12.11%) | 30 (10.08%) | 42 (10.82%) | 24 (9.96%) | 28 (12.84%) |
|  | **NOT Hispanic or Latine** | 390 (81.42%) | 247 (82.89%) | 323 (83.25%) | 200 (82.99%) | 177 (81.19%) |
|  | **Unknown / Not Reported** | 31 (6.47%) | 21 (7.05%) | 23 (5.93%) | 17 (7.05%) | 13 (5.96%) |
| **Race** | **Asian** | 27 (5.64%) | 16 (5.37%) | 21 (5.41%) | 11 (4.56%) | 8 (3.67%) |
|  | **Black or African American** | 30 (6.26%) | 17 (5.7%) | 22 (5.67%) | 16 (6.64%) | 13 (5.96%) |
|  | **More Than One Race** | 25 (5.22%) | 12 (4.03%) | 17 (4.38%) | 11 (4.56%) | 8 (3.67%) |
|  | **Other** | 19 (3.97%) | 11 (3.69%) | 13 (3.35%) | 7 (2.9%) | 7 (3.21%) |
|  | **Unknown / Not Reported** | 13 (2.71%) | 9 (3.02%) | 11 (2.84%) | 6 (2.49%) | 6 (2.75%) |
|  | **White** | 363 (75.78%) | 233 (78.19%) | 303 (78.09%) | 190 (78.84%) | 175 (80.28%) |
| **Sex** | **Female** | 217 (45.3%) | 128 (42.95%) | 175 (45.1%) | 108 (44.81%) | 90 (41.28%) |
|  | **Male** | 262 (54.7%) | 170 (57.05%) | 213 (54.9%) | 133 (55.19%) | 128 (58.72%) |
|  | **Unknown / Not Reported** | 0 | 0 | 0 | 0 | 0 |

**
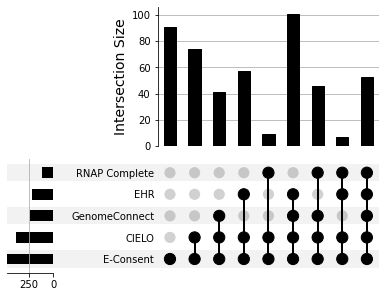
**

**Supplementary Figure 1. Counts and Intersections of Data Sources Available for Each Participants.**

**
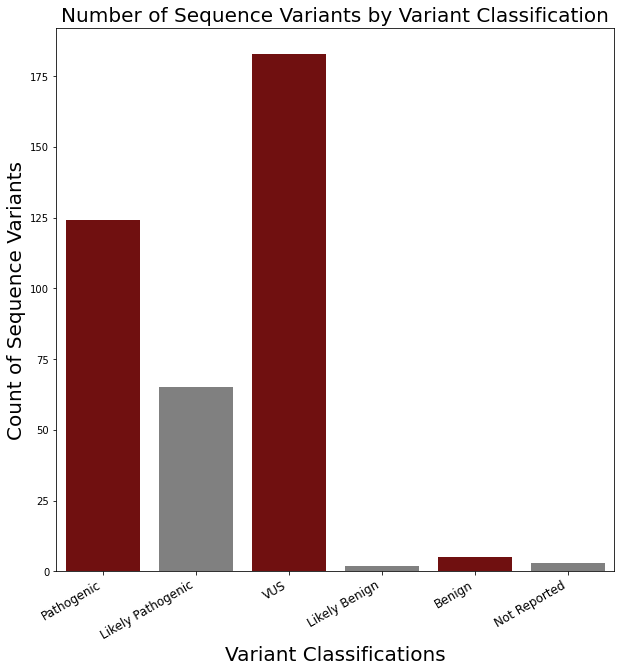
**

**Supplementary Figure 2. Count of Sequence Variants by Variant Classification.**


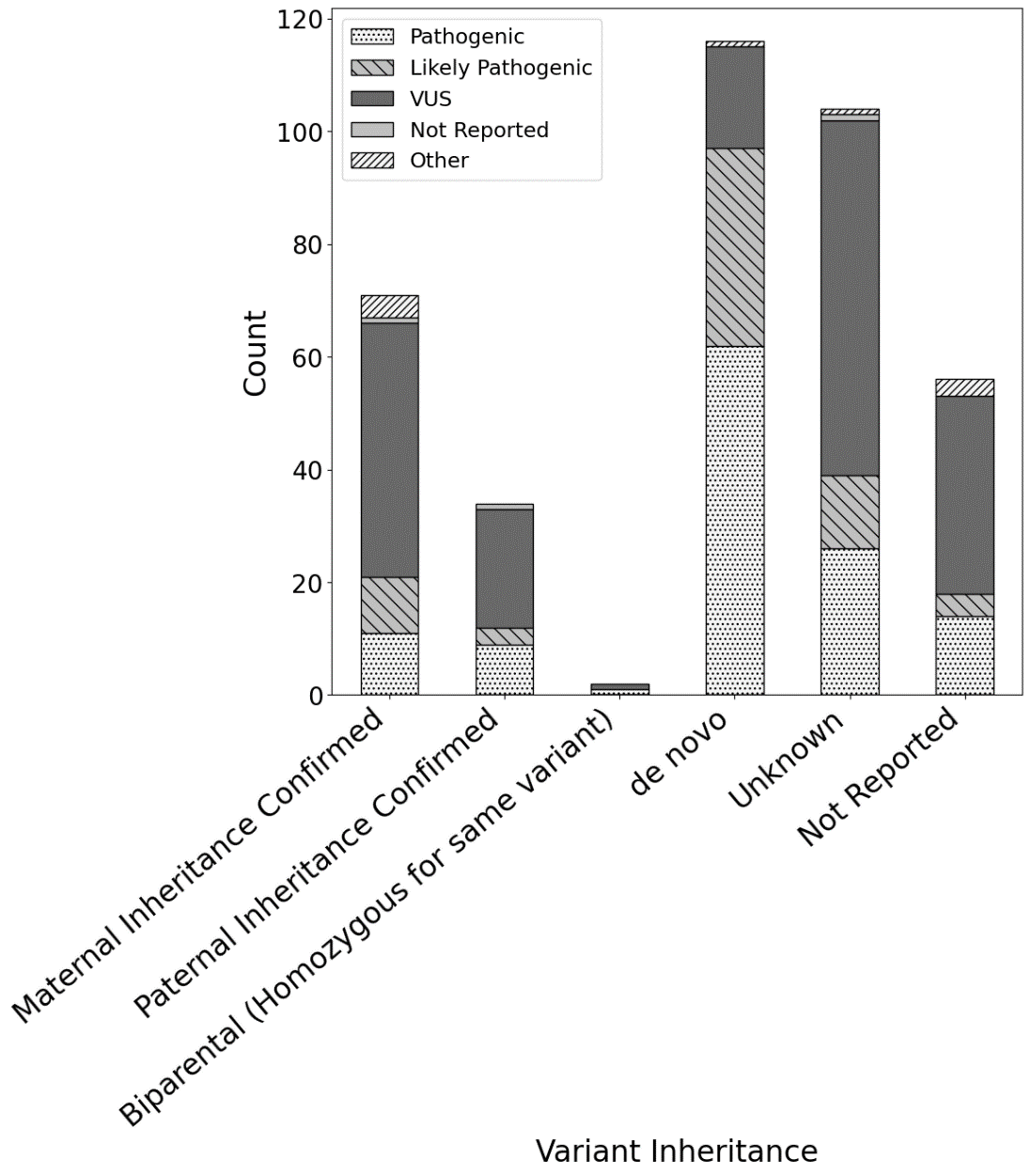


**Supplementary Figure 3. Count of Variant Inheritance by Variant Clinical Classification.** Benign (n = 5) and Likely Benign (n = 2) variants were excluded from analysis to improve visibility of VUS, Likely Pathogenic, and Pathogenic Variants.

**
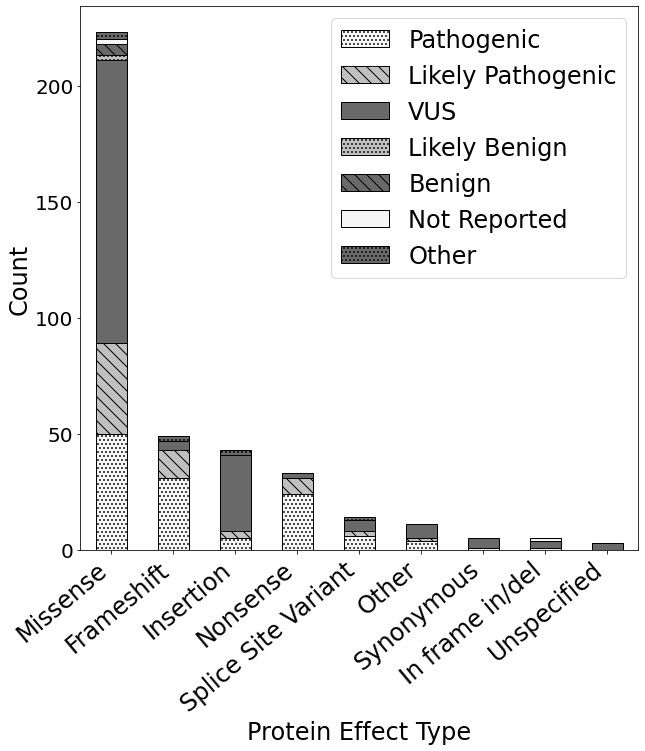
**

**Supplementary Figure 4. Count of Each Protein Effect Type Subset by Variant Classification.**

**
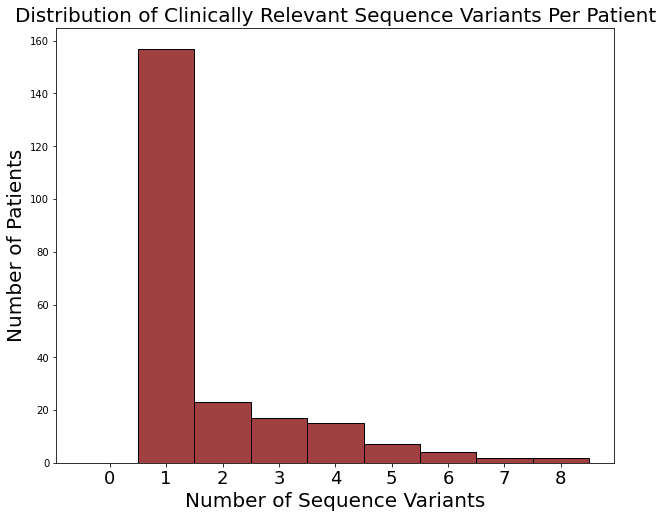
**

**Supplementary Figure 5. Distribution of the Number of Clinically Relevant Sequence Variants per Participant.**

**
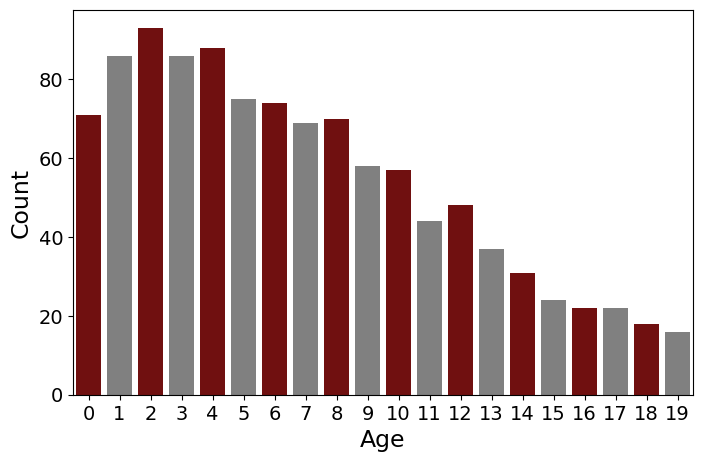
**

**Supplementary Figure 6. Count of Individuals with Diagnosis Information by Age at Encounter.** A participant is counted in each bar for which diagnoses are available in their EHR data at that specified age.

**
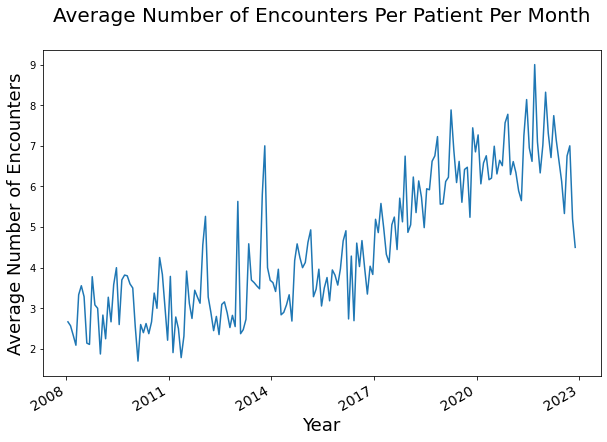
**

**Supplementary Figure 7. Average Number of EHR Encounters per Participant per Month.**

**
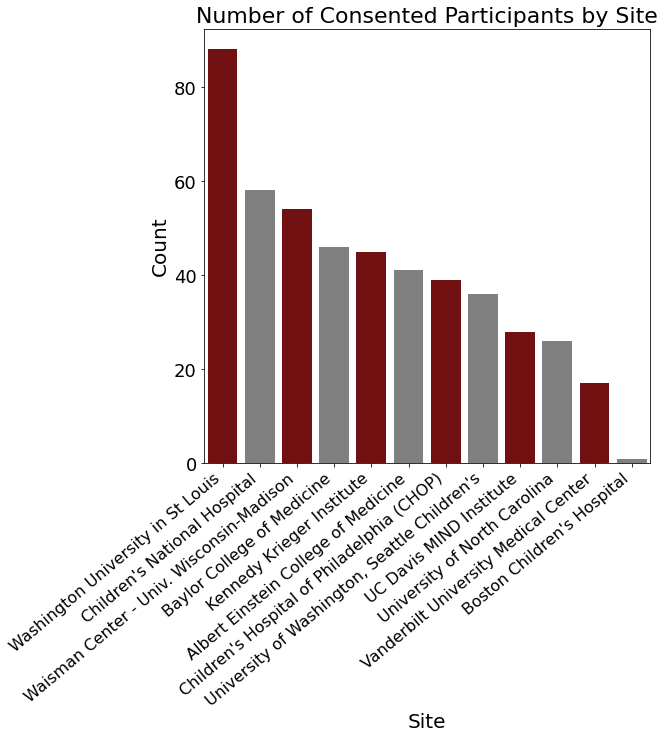
**

**Supplementary Figure 8. Number of Enrolled and Consented Participants by Site.**

**
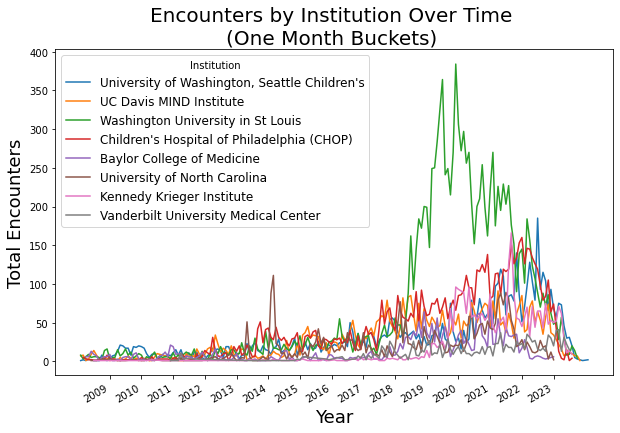
**

**Supplementary Figure 9. Total Number of Monthly Encounters by Institution.**

**
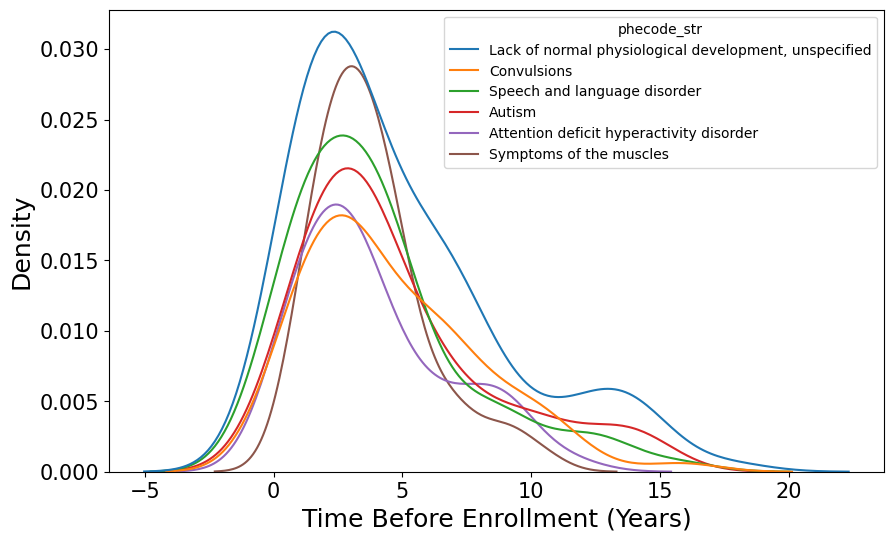
**

**Supplementary Figure 10. Time Observed (in Years) Between the First Phecode Appearance for a Particular Condition in the EHR Data Compared to the BGR Enrollment Date.**

**
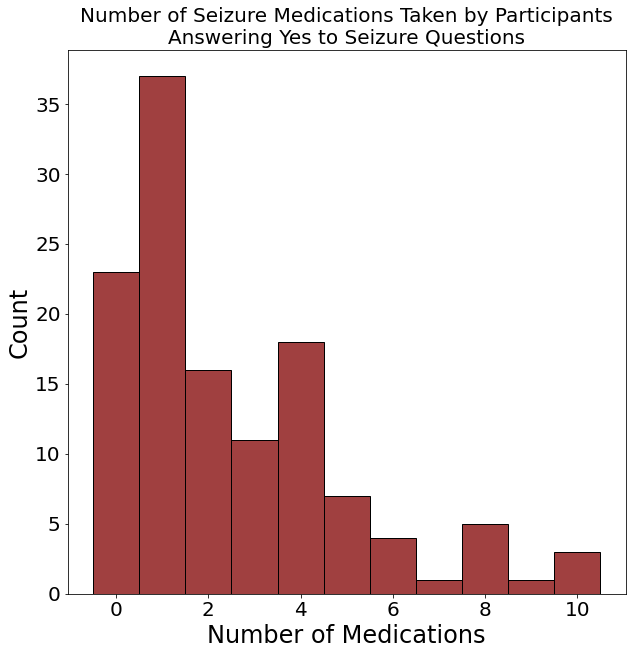
**

**Supplementary Figure 11. Total Number of Seizure Medications Taken by Participants Answering Yes to RNAP Seizure Survey Questions.**
